# Defense Inducers Mediated Mitigation of Bacterial Canker in Tomato through Alteration in Oxidative Stress Markers

**DOI:** 10.3390/microorganisms10112160

**Published:** 2022-10-31

**Authors:** Ruchi Tripathi, Karuna Vishunavat, Rashmi Tewari, Sumit Kumar, Tatiana Minkina, Ugo De Corato, Chetan Keswani

**Affiliations:** 1 Department of Plant Pathology, College of Agriculture, G B Pant University of Agriculture and Technology, Pantnagar 263145, India; 2 Department of Mycology and Plant Pathology, Institute of Agricultural Sciences, Banaras Hindu University, Varanasi 221005, India; 3 Academy of Biology and Biotechnology, Southern Federal University, Rostov-on-Don 44090, Russia; 4 Department of Bioenergy, Biorefinery and Green Chemistry (TERIN-BBC-BIC)-Italian National Agency for the New Technologies, Energy and Sustainable Economic Development (ENEA)-Territorial Office of Bari, Via Giulio Petroni 15/F, 70124 Bari, Italy

**Keywords:** *Cmm*, *Solanum lycopersicum*, ROS metabolism, antioxidant enzymes, defense inducers

## Abstract

The bacterial canker disease of tomato caused by *Clavibacter michiganensis* subsp. *michiganensis* (*Cmm*) has been reported to adversely affect the tomato cultivation in the NE hilly regions of India. Defense inducers such as salicylic acid (SA), isonicotinic acid (INA), benzothiadiazole (BTH) and lysozyme were used as prophylactic and curative sprays at different concentrations to test their efficacy in inducing resistance in tomato plants against *Cmm* under protected conditions. The induced resistance was studied through the alteration in the activities of oxidative stress marker enzymes (PAL, PO, PPO, TPC and PR-2 protein), hydrogen peroxide formation in leaf tissues and lignin accumulation in stem tissues, as well as through the reduction in disease severity under glasshouse conditions. The results of the present study revealed that the enzymatic activity, hydrogen peroxide formation and lignin production were significantly higher in the BTH (500 ppm)-treated leaves than in those observed in the control. The lowest disease incidence was recorded when BTH was applied as a prophylactic spray (27.88%) in comparison to being applied as a curative spray (53.62%), thereby suggesting that a defense inducer, BTH, shows antibacterial activity against *Cmm*, reduces disease incidence severity and induces defense responses in the tomato plant.

## 1. Introduction

Tomato (*Solanum lycopersicum* L.) is one of the most extensively cultivated vegetable crops [1]; however, its cultivation is heavily affected by several plant pathogens which affect the quantity and quality of the produce. The crop is generally affected by different fungal, bacterial, viral and nematode diseases in the hilly region of northern India. Among the bacterial diseases, bacterial wilt (*Ralstonia solanacearum*) and bacterial spot (*Xanthomonas campestris* pv. *vesicatoria*) have been reported [2], often adding to the grower’s losses. While most of the tomato diseases are caused by Gram-negative bacteria, a bacterial disease causing leaf necrosis, wilting and splitting of stem and cankers in the fruits have been observed in recent years, causing damage to the crop in the tomato-growing areas of the northern hilly region. The disease was detected to be bacterial canker caused by the Gram-positive bacterium *Clavibacter michiganensis* ssp. *michiganensis* (Smith) Davis. The world-wide spread of this bacterium is facilitated by contaminated seed stocks, in which a single infected seed in 10,000 can initiate an epidemic [3]. The bacterium reduces the quality and quantity of the product, leading to substantial economic losses both in protected and open field conditions, which may sometimes even lead to complete yield loss [4,5,6,7,8].

Bacterial diseases, including bacterial canker, are expected to become more aggressive in the near future due to climate instability, with devastating effects on basic food-producing areas [9]. Disease management becomes difficult due to the unavailability of resistant cultivars and other effective measures. However, defense inducers or eustressors can play a potential role in reducing the disease’s severity by alleviating the defense response and thus by providing efficient resistance in the plants toward the invading pathogen [10]. These eustressors are also known as elicitors and can be obtained either from a plant, microbe or derived synthetically [11]. The priming of susceptible plants with these elicitors leads to an improvement in plant growth and development [12], and also increases the resistance to biotic stress (plant pathogens), both locally and systemically, by inducing the systemic-acquired resistance (SAR) in plants [13,14,15]. The initiation of SAR is frequently linked with varied defense responses taking place within the cells, viz., pathogenesis-related (PR) proteins, phytoalexins synthesis, reactive oxygen species (ROS) accumulation, boosted action of defense-related enzymes [16] and lignin formation [17]. The pretreatment of tomato seedlings with acibenzolar-S-methyl (ASM) significantly reduces *Cmm* growth and disease severity during the course of the infection [18]. This ASM-mediated enhanced resistance is associated with increased activities of plant peroxidase and chitinase [19]. Additionally, DL-β-amino butyric acid (BABA) treatment remarkably suppresses symptom development caused by *Cmm* via the enhanced peroxidase and phenylalanine ammonia-lyase activities of the host plant [20]. Therefore, the purpose of this study was to evaluate the effective defense inducers so that an alternative to antibiotics can be explored for the management of bacterial canker disease in tomato.

## 2. Materials and Methods

### 2.1. Source of Cmm and Planting Material

The strain *Cmm10* (Genbank Accession No. MH321815) of *C. michiganensis* subsp. *michiganensis*, isolated from Himachal Pradesh, India, was routinely sub-cultured in nutrient broth–glucose–yeast medium (NGY: Nutrient Broth: 8.0 g, Yeast extract: 2.0 g, K_2_HPO_4_: 2.0 g, KH_2_PO_4_: 0.5 g, Glucose: 2.5 g, Agar: 15.0 g, in 1 L of distilled water followed by sterilization at 121 °C, at 15 psi for 15–20 min) was routinely used for the experiments.

Seedlings of susceptible tomato cv. Rohini and US 2853 were grown in 10 cm pots in a soil + sand + vermicompost mix (2: 1: 1) in the glasshouse at 28 ± 2 °C with 68–80% RH with alternate light and dark cycle (10 h: 14 h: dark: light). Leaves, disinfected with 70% ethanol and sterile distilled water (DW) were used for the study.

### 2.2. Preparation and Application of Defense Inducers

Salicylic acid (SA), isonicotinic acid (INA), benzothiadiazole (BTH) and lysozyme were procured from Sigma-Aldrich, Co. (Darmstadt, Germany) and were dissolved in sterile distilled water for the final concentrations of 200 ppm, 500 ppm and 800 ppm. Plants were grouped into five sets for each treatment with ten replicates. Set I: sprayed with SA, INA, BTH and lysozyme (200 ppm); Set II: sprayed with SA, INA, BTH and lysozyme (500 ppm); Set III; sprayed with SA, INA, BTH and lysozyme (800 ppm); Set IV; sprayed with *Cmm*; Set V: sprayed with water. About 200 µL of the prepared concentration was sprayed onto whole seedlings [18] and the seedlings were maintained in glasshouse in the aforementioned conditions. Leaves (1 gm) were collected after two days of treatment to evaluate various biochemical attributes.

### 2.3. Bacterial Strain Inoculation

The bacterial strain *Cmm10* was maintained in NGY at 4 °C. Inoculum was prepared from early log-phase cells of bacterial strain grown in nutrient yeast extract broth at 27 °C on an orbital shaker at 200 rpm for 24 h. Bacteria were then pelleted by centrifugation (twice, each at 3500 rpm for 5 min) and the pellet was rinsed twice by sterilized water and adjusted to the value of 0.06 at OD660 nm, which corresponds to 10^8^ cfu/mL for inoculation. The two youngest leaves were then inoculated by cutting at the tips and dipping them into the bacterial suspension [21].

### 2.4. Sample Preparation for Enzymatic Activity Determination

#### 2.4.1. Sample Collection

The samples were taken from the seedlings sprayed with four defense inducers at three different concentrations (200 ppm, 500 ppm and 800 ppm), at two different durations for biochemical analysis after 48 h in all the sets of experiment to assess the change in enzymatic activity. Leaf tissues were taken at the actual site of inoculation with *Cmm* in the case of inoculated plants and from sites similar to those on inoculated leaves in the case of the control plants. These leaf tissues were stored in a deep freezer (−80 °C) until used for biochemical analysis.

#### 2.4.2. Biochemical Analysis

##### Polyphenol Oxidases (PPOs) Assay (EC 1.14.18.1)

Leaf samples (0.1 g) were homogenized in 2 mL of ice-cold phosphate buffer (0.1 M/L) at pH 6.5 and the homogenate was centrifuged at 16,000 rpm for 30 min at 4 °C. The supernatant thus obtained was used directly in the enzyme assay. The reaction mixture for this assay contained 0.4 mL catechol, the substrate for PPOs estimation (1 mM/L) in 3 mL of (0.05 M/L) sodium phosphate buffer at pH 6.5 and 0.4 mL enzyme extract, whereas the reaction mixture without the enzyme extract served as control. The change in absorbance was recorded at 405 nm [22] and the PPO enzyme activity was expressed as change in OD min/mg/FW.

##### Phenylalanine Ammonia Lyases (PALs) Assay (EC 4.1.3.5)

For PALs assay, 0.1 g leaf samples from each treatment were homogenized in 2 mL of sodium borate buffer (0.1 M/L; pH 7.0; 4 °C) containing (1.4 mM/L) 2-mercaptoethanol. Homogenate was centrifuged at 16,000 rpm at 4 °C for 15 min and the supernatant was used as enzyme source. The reaction mixture containing 0.2 mL enzyme extract, 0.5 mL (0.2 M/L) borate buffer at pH 8.7 and 1.3 mL of water was prepared, and the reaction was initiated by the addition of 1 mL L-phenylalanine (0.1 M/L at pH 8.7) followed by an incubation at 32 °C for 30 min. The termination of the ongoing reaction was achieved by pouring 0.5 mL of trichloroacetic acid (TCA, 1 M/L) into the reaction mixture. The measurement of PALs activity was conducted by estimating trans-cinnamic acid formation at 290 nm and was expressed as µmol/min/g fresh weight (FW) TCA [23].

##### Peroxidases (POs) Assay (EC 1.11.1.7)

Peroxidases assay was performed by homogenizing leaf samples (0.1 g) in 2 mL of ice-cold phosphate buffer (0.1 M/L), with a pH of 7.0 at 4 °C, centrifuged at 16,000 rpm at 4 °C for 15 min, and the supernatant was used as enzyme source. Pyrogallol (0.05 M/L) (1.5 mL), enzyme extract (0.05 mL) and 0.5 mL H_2_O_2_ (1% *v*/*v*) was used as reaction mixture. The changes in the absorbance at 420 nm were recorded after 30 s intervals for 3 min and the enzyme activity was expressed as change in the U/min/g FW [24].

##### Total Phenol Content (TPC) Assay

For estimation of total phenol content, the leaf tissues (0.1 g) dished in 5 mL ethanol (95%) were placed at 0 °C for 48 h. Each sample was homogenized and centrifuged at 10,000 rpm for 10 min. Reaction mixture containing 1 mL of 95% ethanol, 5 mL of autoclaved distilled water and 0.5 mL of 50% Folin–Ciocalteau reagent was added to 1 mL of the supernatant and well shaken. Then, 1 mL of 5% sodium carbonate was added after 5 min and the reaction mixture was incubated at room temperature for an hour. The absorbance of the developed color was recorded at 725 nm [25]. Standard curves were prepared for each assay using different concentrations of gallic acid (GA) in 95% ethanol. Absorbance values were converted to mg GA equivalents (GAEs) g^−1^ FW.

##### PR2 (β-1,3-Glucanase) Protein Assay

The β-1,3-glucanase activity was assayed by the laminarin-dinitrosalicylic acid method [26]. The reaction mixture consisted of 62.5 µL of 4% laminarin and 62.5 µL of enzyme extract. The reaction mixture was carried out at 40 °C for 10 min. The reaction was stopped, 375 µL of dinitrosalicylic acid was added and the reaction was heated for 5 min in boiling water, then vortexed and the absorbance was measured at 500 nm. The enzyme activity was measured as µg glucose released in min^−1^ mg^−1^ protein.

### 2.5. Histochemical Analysis

#### 2.5.1. Hydrogen Peroxide Production

The histochemical analysis of hydrogen peroxide (H_2_O_2_) was performed by DAB (3, 3-diaminobenzidine) resulting in a reddish-brown staining [27]. Leaf discs were immersed in DAB solution (1 mg ml^−1^; pH 7.5) and incubated in the dark for 20 h at room temperature for qualitative estimation of H_2_O_2_. The leaf discs were then boiled in 15 mL solution containing absolute ethanol and lactophenol (2:1) for 5 min and then rinsed with ethanol (1 mL of 50%) twice and finally examined under light microscope (Nikon DS-fi1, Tokyo, Japan) to estimate H_2_O_2_ production in leaf tissues.

#### 2.5.2. Lignification

Transverse stem sections from each treatment were examined. The stem sections were fixed in 95% (*v*/*v*) ethanol mounted on a slide in a solution of saturated aqueous phloroglucinol in 20% hydrochloric acid and observed with light microscope (Leica (Wetzlar, Germany) DM2500). Positive lignin staining was indicated by red-violet coloration of the tissue [28].

### 2.6. Assessment of Defense Inducers Efficacy on Disease Incidence under Protected Conditions

Tomato seedlings (Cv. Rohini and US2853) were maintained in the glasshouse in the aforementioned conditions and were treated with defense inducers in two sets at the fifth week stage. In Set I: the curative spray of the defense inducers was given at 200 ppm, 500 ppm and 800 ppm, and in Set II: prophylactic spray of the defense inducers was given at equal concentrations to determine the most efficient concentration and spray duration for disease management. For the inoculation of healthy plants with *Cmm*, the two youngest leaves at the fifth week stage of the plants were inoculated by cutting them at the tips and dipping them into the bacterial suspension (10^8^ cfu) [21]. The tomato plants treated only with water served as negative control and plants inoculated only with *Cmm* served as positive control. Disease progress was monitored until 21 days after inoculation. Disease incidence (percentage of plant infection) was recorded by using the standard formula. Fifty plants were used for each set of concentration and the experiments were replicated thrice.

Disease incidence (%) = (Number of diseased plants in the plot/Total number of plants in the plot) × 100.

### 2.7. Statistical Packages

Data obtained were analyzed by Statistical Package for Social Science (SPSS) version 26.0 for Windows (SPSS, Chicago IL, USA). Descriptive statistics (mean and standard deviation) analysis of variance with means separated using Tukey’s test and level of significance were considered as *p*≤ 0.05. The figures were generated in Prism 9.0.1 (GraphPad Software, La Jolla, CA, USA).

## 3. Results

### 3.1. Effect on Peroxidase Activity

**Figure 1:**
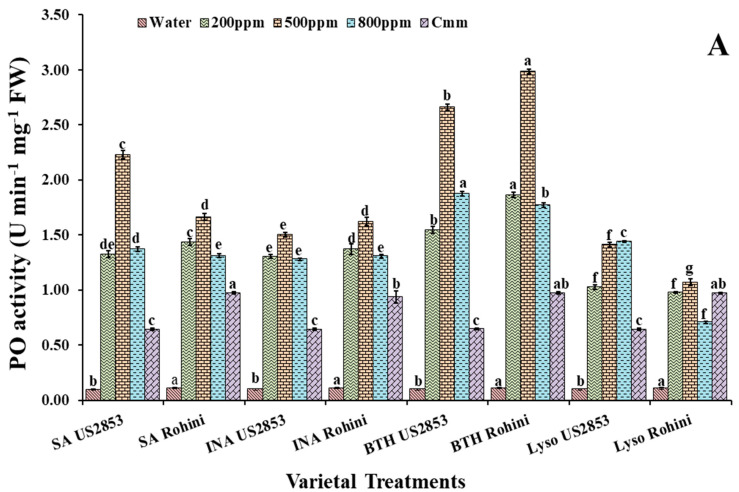

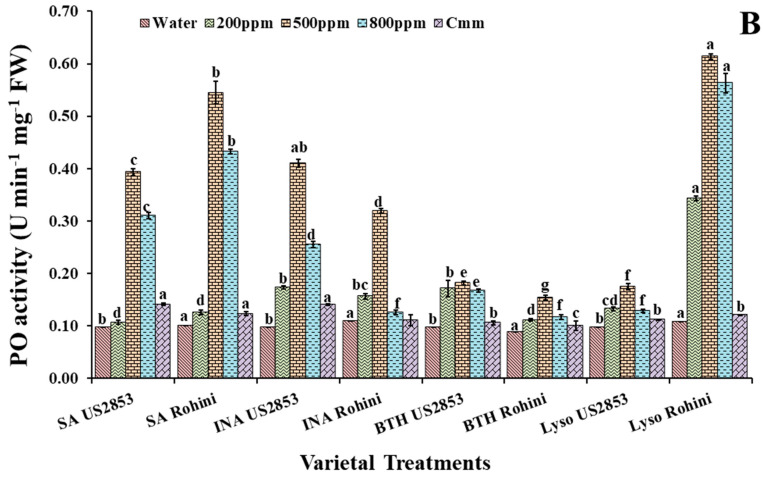
Peroxidase activity in tomato plants at 48 h after (**A**) prophylactic and (**B**) curative spray of SA, INA, BTH and lysozyme in cultivars US2853 and Rohini. Vertical bars indicate standard deviations of the means. Different letters indicate significant differences among treatment results taken at the same time interval according to Tukey’s test at *p* ≤ 0.05.

POx activity was significantly higher in the plants sprayed with defense inducers at 500 ppm in both varieties, followed by 200 ppm (Figure 1A). However, at an 800 ppm concentration, a phytotoxicity symptom was observed in some plants. The enzymatic activity in the plants sprayed only with the pathogen was near to that of a 200 ppm concentration and the least enzymatic activity was observed in the plants sprayed only with water.

When the prophylactic spray of the defense inducers was applied, the maximum enzymatic activity was observed in the plant sprayed with BTH followed by SA, INA and lysozyme. Enzymatic activity was lesser in US2853 in comparison to Rohini. A significant elevation of the PO activity was observed in the varieties Rohini and US2853, which received the BTH foliar spray at a 500 ppm concentration (2.97, 2.68) (*p* < 0.05), followed by the plants sprayed with defense inducers at 200 ppm and 800 ppm. The least enzymatic activity was observed in cultivar US2853 (0.098), sprayed only with water, followed by the plants inoculated only with the pathogen (0.642) in cultivar US2853.

Enzymatic activity reached the maximum in the plants treated with SA in both the cultivars (1.78, 0.925; *p* ≤ 0.05), respectively, at a 500 ppm concentration followed by 200 ppm and 800 ppm when the curative spray of defense inducers was applied (Figure 1B). However, a rise in enzymatic activity was also observed in the Rohini cultivar plants receiving a curative spray of lysozyme. The least enzymatic activity was observed in cultivar US2853 (0.098), sprayed only with water, followed by the plants only inoculated with the pathogen (0.642) in cultivar US2853.

### 3.2. Effect of Defense Inducers on Phenylpropanoid Activity

#### 3.2.1. PAL Activity

**Figure 2:**
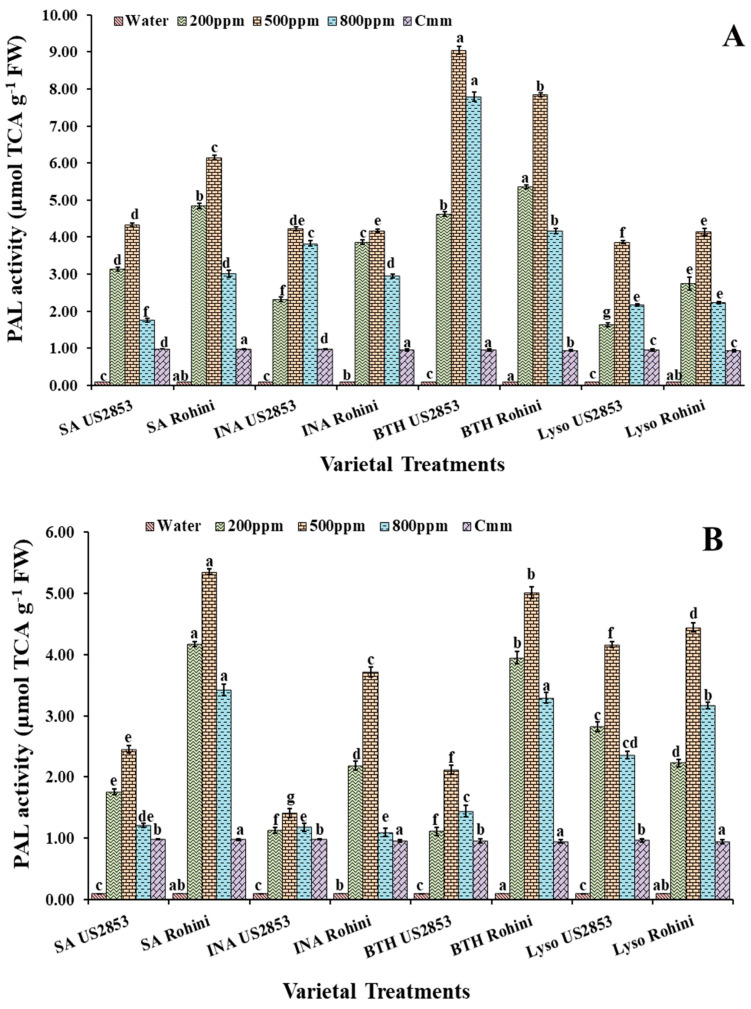
PAL activity in tomato plants at 48 h after (**A**) prophylactic and (**B**) curative spray of SA, INA, BTH and lysozyme in cultivars US2853 and Rohini. Vertical bars indicate standard deviations of the means. Different letters indicate significant differences among treatment results taken at the same time interval according to Tukey’s test at *p* ≤ 0.05.

The maximum PAL activity was observed in the plants given the prophylactic spray with BTH in the cultivar Rohini followed by US2853 (12.02, 7.82, *p* ≤ 0.05) at a 500 ppm concentration (Figure 2A). The second highest enzymatic activity was observed in the plants sprayed with SA (6.14, 4.13, *p* ≤ 0.05), INA and lysozyme. Amongst the four treatments, the least effective treatment was that of lysozyme at 800 ppm (2.23, 2.17) (*p* ≤ 0.05), in cultivars Rohini and US2853, respectively.

Within the plants receiving the spray of defense inducers after the inoculation of the pathogen, the highest enzymatic activity was observed in the plants treated with SA at 500 ppm (5.37, 2.46; *p* ≤ 0.05) (Figure 2B) followed by BTH and lysozyme at 500 ppm concentrations. The least enzymatic activity amongst the treatment was observed in the plants treated with INA (3.71, 1.45; *p* ≤ 0.05) in cultivars Rohini and US2853, respectively.

#### 3.2.2. Total Phenolic Content

**Figure 3:**
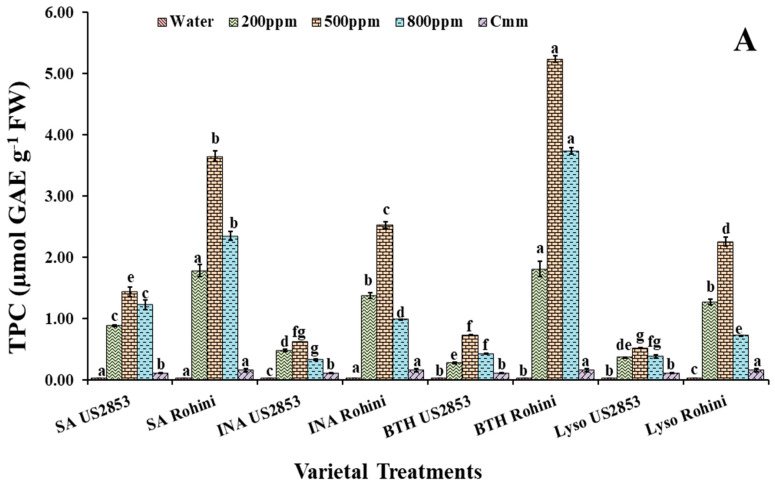

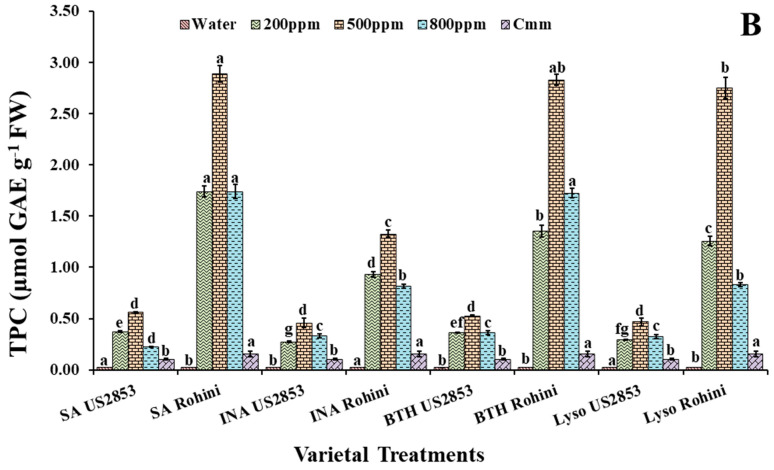
Total phenols in tomato plants at 48 h after (**A**) prophylactic and (**B**) curative spray of SA, INA, BTH and lysozyme in cultivars US2853 and Rohini. Vertical bars indicate standard deviations of the means. Different letters indicate significant differences among treatment results taken at the same time interval according to Tukey’s test at *p* ≤ 0.05.

The treatments with defense inducers (SA, INA, BTH and lysozyme) elevated the total phenolic content significantly in plants receiving the prophylactic spray of defense inducers in comparison to the control. However, phenolic content was significantly higher in the plants sprayed with BTH at a 500 ppm concentration in the cultivars Rohini and US2853 (5.18, 0.726, *p* < 0.05), respectively. An elevated phenolic content was observed in the SA-treated plants followed by the INA-treated plants, and the minimum phenolic content amongst the four treatments was observed in the plants treated with lysozyme (2.18, 0.515, *p* ≤ 0.05) (Figure 3A) in cultivar Rohini.

It was observed that in the plants receiving the curative spray of defense inducers (SA, INA, BTH and lysozyme), at three varied concentrations (200 ppm, 500 ppm and 800 ppm), the phenolic content was higher in plants sprayed with SA in both the cultivars viz., Rohini and US2853 (3.92, 0.559, *p* ≤ 0.05) (Figure 3B), followed by the plants treated with BTH and lysozyme. The minimum phenolic content amongst the four treatments was observed in the plants treated with INA (1.36, 0.417; *p* ≤ 0.05).

#### 3.2.3. Polyphenol Oxidase Activity

**Figure 4:**
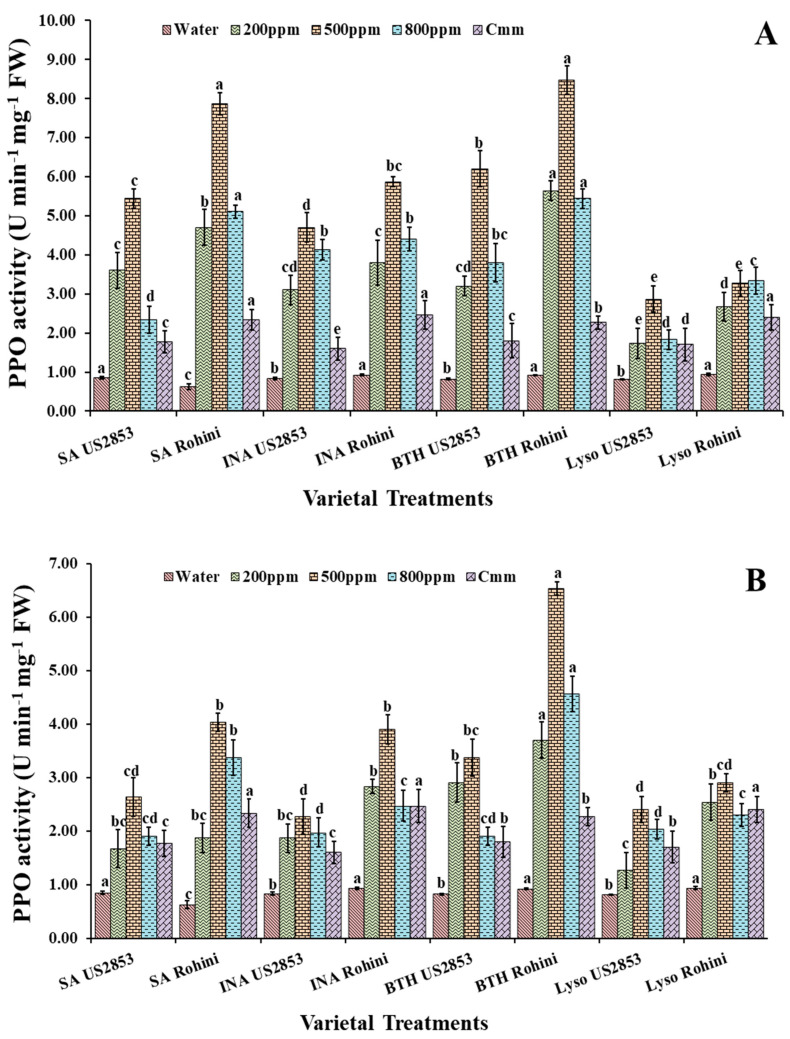
Polyphenol oxidase activity in tomato plants at 48 h after (**A**) prophylactic and (**B**) curative spray of SA, INA, BTH and lysozyme in cultivars US2853 and Rohini. Vertical bars indicate standard deviations of the means. Different letters indicate significant differences among treatment results taken at the same time interval according to Tukey’s test at *p* ≤ 0.05.

The prophylactic spray of defense inducers showed a significant increase in the PPO activity in comparison to the positive and negative controls. The highest PPO activity was observed in the plants sprayed with BTH at 500 ppm in the cultivar Rohini followed by the cultivar US2853 (81.68, 61.30, *p* < 0.05) (Figure 4A). The least enzymatic activity was observed in the plants treated with lysozyme (31.82, 19.57, *p* ≤0.05) at the same concentration. The minimum enzymatic activity was observed in the plants sprayed only with water.

The findings indicate that, while receiving the treatment of defense inducers after inoculation with the pathogen, the maximum enzymatic activity was observed in the plants treated with SA in both the cultivars viz., Rohini and US2853 (67.39, 38.3, *p* < 0.05) (Figure 4B) at a 500 ppm concentration followed by the enzymatic activity at 200 ppm and 800 ppm. The second highest enzymatic activity was observed in the plants treated with BTH followed by INA and lysozyme with similar concentration patterns. The minimum enzymatic activity was observed in the plants sprayed only with water followed by the plants inoculated only with the pathogen.

### 3.3. Effect of Defense Inducers on β-1,3-Glucanases (PR-2 Protein) Activity

**Figure 5:**
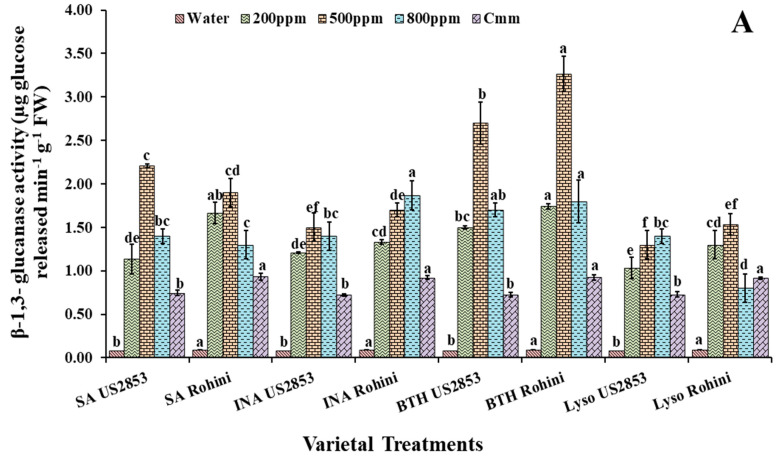

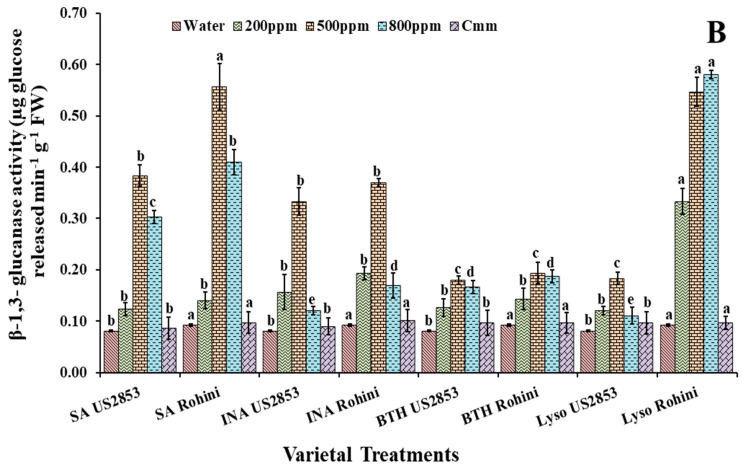
β-1,3-glucanases activity in tomato plants at 48 h after (**A**) prophylactic and (**B**) curative spray of SA, INA, BTH and lysozyme in cultivars US2853 and Rohini. Vertical bars indicate standard deviations of the means. Different letters indicate significant differences among treatment results taken at the same time interval according to Tukey’s test at *p* ≤ 0.05.

It is also observed that in the plants receiving the prophylactic spray of the defense inducers, the level of β-1,3-glucanases was significantly higher when treated with BTH at a 500 ppm concentration (3.28, 2.75, *p* ≤ 0.05). The second highest enzymatic activity was observed in the plants treated with SA followed by the plants treated with INA. The least enzymatic activity was observed in the plants receiving the lysozyme treatment (1.09, 1.42, *p* ≤ 0.05) (Figure 5A) in cultivars Rohini and US2853, respectively.

Studies have indicated that in the plants receiving the treatments of defense inducers at 200 ppm, 500 ppm and 800 ppm for the four-defense inducer, after the inoculation of the pathogen, the maximum activity in the cultivar Rohini was observed at the lysozyme 500 ppm concentration (0.672, *p* ≤ 0.05) (Figure 5B), while in cultivar US2853 the maximum enzymatic activity was found at the SA 500 ppm concentration (0.393, *p* ≤ 0.05). The minimum PR-2 protein activity was observed in the tomato plants treated with BTH (0.11, 0.12, *p* ≤ 0.05) in cultivars Rohini and US2853, respectively.

### 3.4. Effect of Defense Inducers on Hydrogen Peroxide Generation

The formation of hydrogen peroxide in the leaves can be observed as the reddish-brown coloration of the leaf tissue easily visible to the naked eye. The leaf tissue sections undergo the DAB staining, which is distributed uniformly throughout the leaves. A simple test required for ensuring the peroxidase activity involves the exposure of the plant tissues to DAB and H_2_O_2_. DAB polymerization was studied in treated and untreated leaves after 48 h of incubation and the effect of the defense inducers and pathogen inoculation on peroxidase activity was determined on leaf tissues.

**Figure 6:**
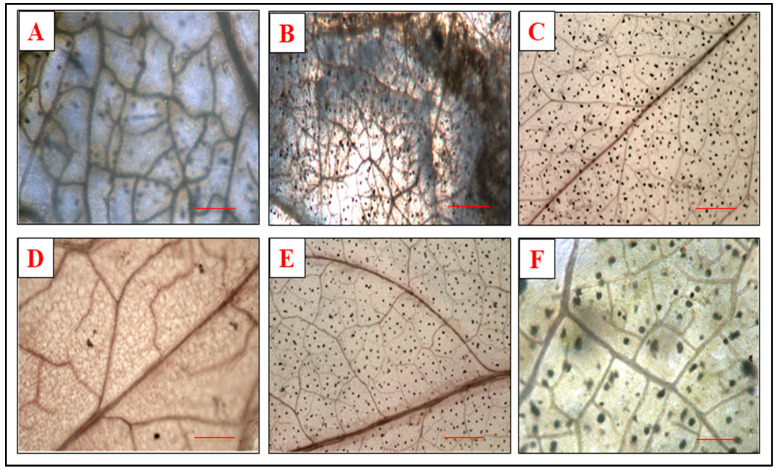
Visualization of hydrogen peroxide formation in the leaves of the plants receiving the prophylactic spray of defense inducers: (**A**) positive control (PC), (**B**) negative control (NC), (**C**) salicylic acid (SA), (**D**) isonicotinic acid (INA), (**E**) benzothiodiazole (BTH) and (**F**) lysozyme at 500 ppm after 48 h of treatment.

It was observed that in the tomato leaves, when given the prophylactic spray of the defense inducers (Figure 6), the peroxidase level was higher at 48 h after treatment in plants sprayed with BTH at a 500 ppm concentration followed by the plants sprayed with SA and INA. The minimum hydrogen peroxide production was observed in the plants treated with lysozyme before pathogen inoculation.

### 3.5. Effect of Defense Inducers on Lignin Deposition

**Figure 7:**
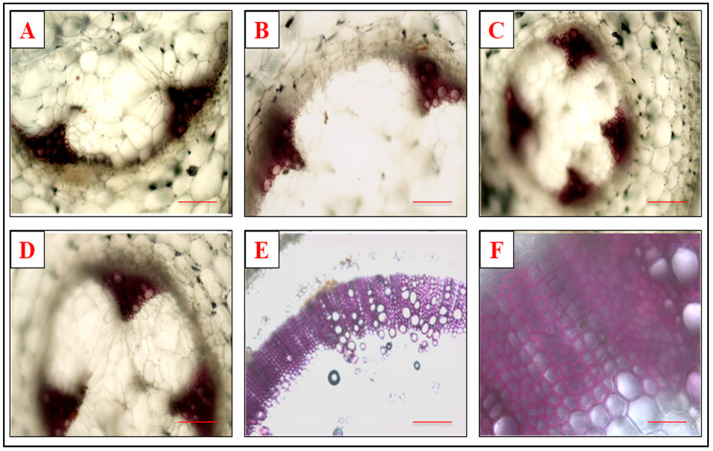
Lignin formation in the xylem vessels of the tomato plants’ stem receiving the prophylactic treatment of defense inducers ((**A**) SA, (**B**) INA, (**C**) BTH, (**D**) lysozyme, (**E**) positive control and (**F**) negative control) at 500 ppm after 48 h of treatment.

A significant variation in the transverse section of the stem from different treatments was observed through histochemical staining. The tomato plants were given the prophylactic treatments of the defense inducers (Figure 7) and were then screened for lignin production at 48 h after the treatment by cutting the transverse section of the stem 1 cm above the point of inoculation. It was observed that lignin content was the highest in the plants sprayed with SA at 500 ppm concentrations, followed by the plants sprayed with BTH and INA. The least lignin content was observed in the plants treated with lysozyme before pathogen inoculation.

### 3.6. Assessment of Defense Inducers Efficacy on Disease Incidence Management and Disease Severity under Protected Conditions

**Figure 8:**
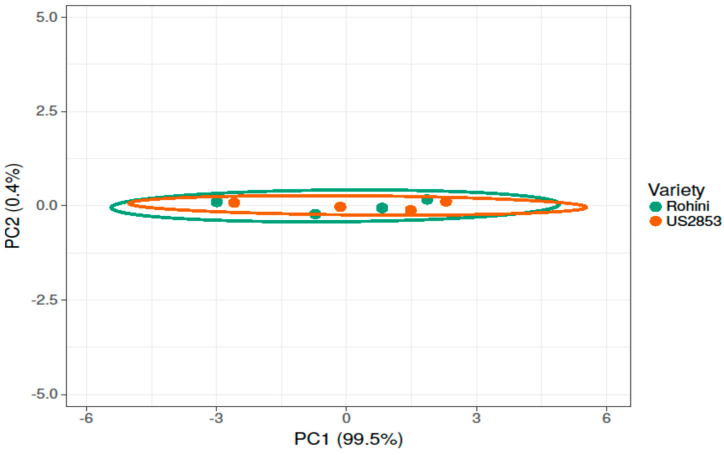
Principal component analysis depicting correlation and significance between defense inducers used at different concentrations and varieties Rohini (moderately resistant) and US 2853 (susceptible check) during prophylactic spray. The analysis was generated using the ClustVis website, https://biit.cs.ut.ee/clustvis/ (accessed on 9 July 2022).

**Figure 9:**
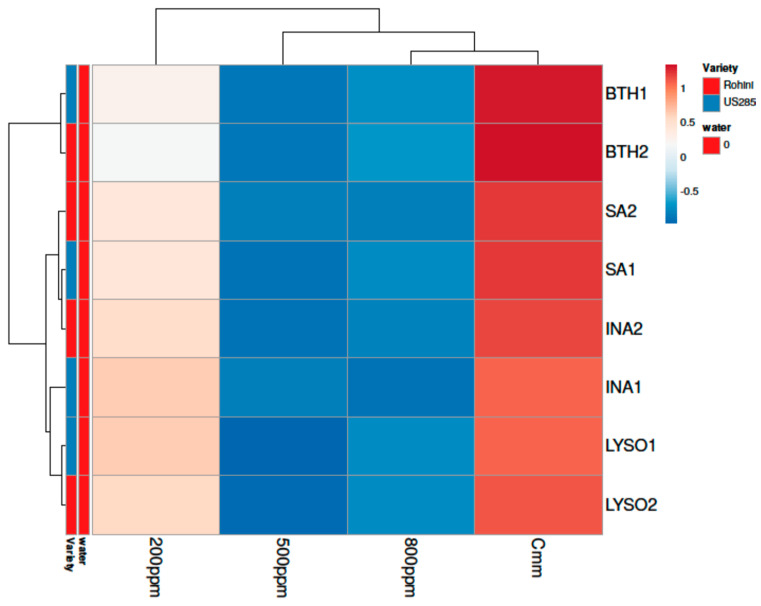
Clustering and heatmap analysis of effect of defense inducers on % disease incidence during prophylactic spray. The rows express the individual defense inducers concentrations and the columns indicate the defense inducers used in the study viz., lysozyme (LYSO), benzothiodiazole (BTH), isonicotinic acid (INA) and salicylic acid (SA). Here, 1 and 2 indicate Variety 1 (US2853) and Variety 2 (Rohini), respectively. Herein, untreated and uninoculated seedlings (water only) served as negative control while untreated and inoculated seedlings (*Cmm* only) served as positive control. Lower numerical values are the blue color, whereas higher numerical values are the red color. Map was generated by using the ClustVis website, https://biit.cs.ut.ee/clustvis/ (accessed on 9 July 2022). The results are expressed as averages of three replications.

While comparing the timings of application and the most efficient doses of defense inducers for effective disease management, it was found that the disease incidence was at its minimum in BTH when applied before pathogen inoculation (27.88%) and it was at its highest in lysozyme (49.72%) with the prophylactic spray at 500 ppm (Figure 8 and Figure 9).

**Figure 10:**
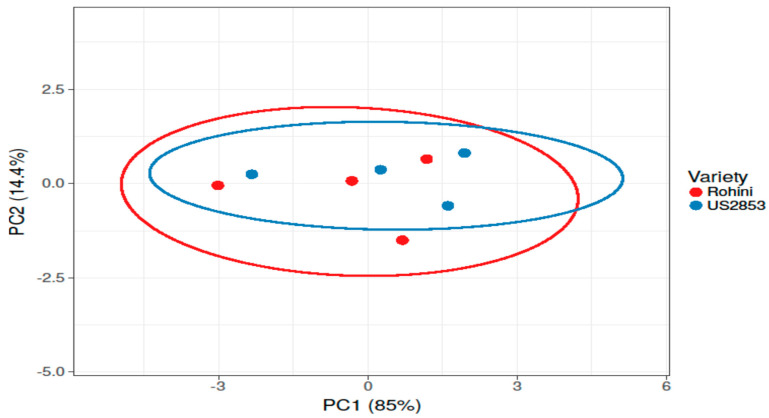
Principal component analysis depicting correlation and significance between defense inducers used at different concentrations and varieties Rohini (moderately resistant) and US 2853 (susceptible check) during curative spray. The analysis was generated by using the ClustVis website, https://biit.cs.ut.ee/clustvis/ (accessed on 9 July 2022).

**Figure 11:**
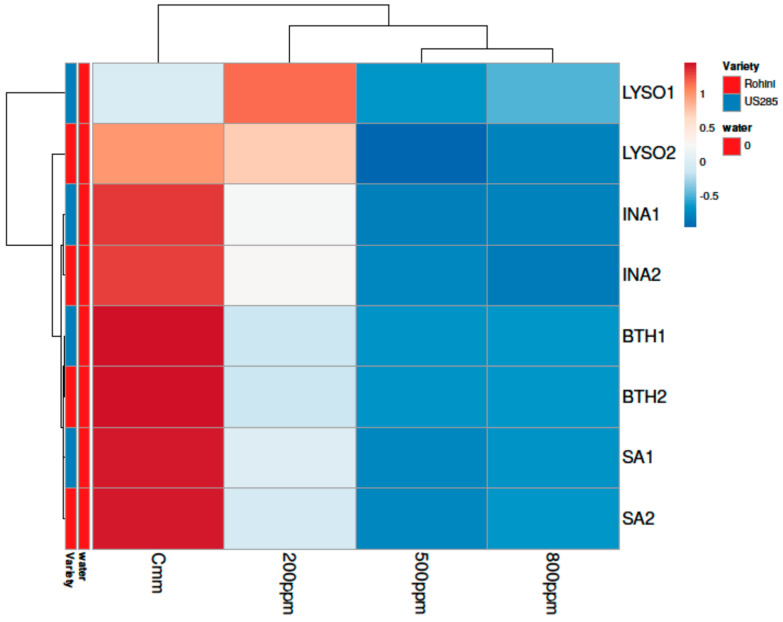
Clustering and heatmap analysis of effect of defense inducers on % diseases incidence during curative spray. The rows express the individual defense inducers concentrations and the columns indicate the defense inducers used in the study viz., lysozyme (LYSO), benzothiodiazole (BTH), isonicotinic acid (INA) and salicylic acid (SA). Here, 1 and 2 indicate Variety 1 (US2853) and Variety 2 (Rohini), respectively. Herein, untreated and uninoculated seedlings (water only) served as negative control while untreated and inoculated seedlings (*Cmm* only) served as positive control. Lower numerical values are the blue color, whereas higher numerical values are the red color. Map was generated by using the ClustVis website, https://biit.cs.ut.ee/clustvis/ (accessed on 9 July 2022). The results are expressed as averages of three replications.

When the plants received the treatments with defense inducers after the inoculation of the pathogen, the treatment exhibiting the lowest disease incidence was BTH (53.62%) and the treatment exhibiting the highest disease incidence was INA (62.94%) at 500 ppm (Figure 10 and Figure 11).

The application of lysozyme at a 500 ppm concentration before or after the inoculation did not exhibit much difference in disease incidence; however, in the case of the other three defense inducers, the prophylactic spray was more effective in the management of the pathogen at 500 ppm. A decline in the percentage of disease incidence was observed at 800 ppm as well, but the treated plants also showed phytotoxic symptoms, making this concentration not suitable for disease management.

## 4. Discussion

The activation of the antioxidant network and the phenylpropanoid pathway are the primary steps taken by the plant as management of biotic stress [29]. SAR development is found to be connected with the varied cellular defense responses, such as PR protein synthesis, formation of phytoalexins and buildup of reactive oxygen species (ROS), fast changes in cell wall composition and significant increases in the concentration and activity of defense-related enzymes [16]. In this study, the dense inducer-treated pathogen-inoculated plants showed an elevation in their enzymatic and phenylpropanoid activities in comparison to the positive and negative controls. The studies exploring active oxygen species (AOS) during SAR expression showed ample evidence that AOS, H_2_O_2_ in particular, execute numerous significant functions in early defense responses of the plant against pathogens, and that these responses generally include mechanisms such as antimicrobial action, lignin formation, phytoalexin production and induction of SAR [17]. AOS may also render deleterious effects on cell health. O_2_, OH and H_2_O_2_ are some of the ROS generally produced under stress conditions [30], acting as attacking oxidizing species that can rapidly affect all types of bio-molecules and easily damage them. Oxygen radical detoxifying enzymes such as catalase, peroxidase and superoxide dismutase (SOD), as well as nonenzymatic antioxidants such as ascorbate peroxidase and glutathione-S-transferase (GST) [31], play an important role in protecting the plant cells from the damage caused due to increased ROS generation [32].

The pre-treatment of plants with different biotic (pathogens and insect pests) and abiotic (chemicals) inducers induces plant resistance which helps defend the plants against subsequent attacks [33,34,35]. The plant phytohormones induce plant defense against many biotic and abiotic stresses. Salicylic acid is an important and well-studied endogenous plant growth regulator that generates a wide range of metabolic and physiological responses in plants involved in plant defense, in addition to their impact on plant growth and development [36,37]. In the current study, the leaf extract analysis showed an increase in the PO accumulation in all the treatments in comparison to the negative and positive controls. SA also activates the generation of ROS and other defensive processes such as hypersensitive response and cell death [38]. The simultaneous inclusion of phenolic compounds in the cell wall during incompatible plant–microbe/elicitor interactions can be associated with an increase in POX activity. The enzyme POXs are supposed to catalyze the last few steps of the lignification pathways in the tomato. A low activity of POX in plants treated with defense inducers at higher concentrations may be due to the phytotoxicity experienced by the plant at higher concentrations [39].

PAL plays an important role in plant defense; it is involved in the biosynthesis of salicylic acid, an essential signal involved in plant systemic resistance. The enhanced enzymatic activity of PAL is the foremost response in a number of plant species to pathogen challenges and is very much associated with resistance [40]. An alleviation in the PAL activity by addition of salicylic acid to *Saussurea medusa* cell cultures at a concentration of 20 μM [41] and a reduction in severity of *Lasidiploidia theobromae* [42] were reported, due to the priming of tea plants with BTH before inoculation with *L. theobromae* after an increase in PAL activity. In the present study, PAL activity was at its highest in the pathogen-inoculated defense-inducers-treated plant as compared to the negative and positive controls.

The stress posed on the plants by varied biotic and abiotic stress inducers can be defended by phenolic compounds [33,43,44]. The physiology of plants and their displayed metabolism can be altered by the oxidation of phenols that produce many defensive compounds, which help the plant in surviving against different stresses either directly or through diverse plant-signaling pathways [45]. Furthermore, ROS such as superoxide anion, hydroxide radicals, H_2_O_2_ and singlet oxygen produced by the oxidation of phenols activate plant defense enzymes [46,47]. PPOs can be considered as one of the most important enzymes involved in plant defense against many biotic and abiotic stresses [33]. Defense inducers such as jasmonic acid, SA and ethylene have been observed to stimulate such enzymes in plants [48]. An increase in the enzymatic activities and phenylpropanoid syntheses was also observed in the Bacillus cereus-treated, *Cmm*-infected tomato plant [49]. The result observed in the present study is in agreement with the findings of various researchers, wherein an elevation in the phenolic content and PPO activity was observed in the defense-inducer-treated pathogen-inoculated plant as compared to the positive and negative controls.

The PR proteins β-1,3-glucanases (PR-2) and chitinases (PR-3) have been recognized to possess enzymatic activities, including direct antimicrobial activity by degrading microbial cell wall components. The enzymatic activities of these proteins lead to the breakdown of the pathogen and/or plant cell wall components which act as elicitors to plant defense responses [50]. Chitinase enzymes have been reported to have lysozymal activity leading to bacterial cell wall hydrolyses in a few plants [51,52]. The PR genes expression and the connected accretion of the encoded PR proteins have often been considered as the molecular basis of induced resistance. SA, JA and ET stimulate the production of antimicrobial compounds such as phytoalexins and pathogenesis-related (PR) proteins [53] that eventually initiate a hypersensitive response (HR) that causes infected cell death and pathogen containment [52,54]. In the present study, an elevation in the β-1,3-glucanases activity was also observed in the defense-inducer-treated pathogen-inoculated plant as compared to the positive and negative controls.

Salicylic acid and its functional analogous have also been reported to increased H_2_O_2_ levels in treated tobacco leaves [55]. H_2_O_2_ plays a vital role not only in stimulating hypersensitive cell death, but also in restricting the spread of cell death by inducing the expression of cell-protecting genes in surrounding cells [31]. In the present study, a higher accumulation of H_2_O_2_ was also observed in plants treated with defense inducers, and similar histochemical studies carried out by [34] also clearly showed that defense inducers such as BTH and ASM induce H_2_O_2_ accumulation in bean plants. The elevated levels of H_2_O_2_, which result from the inhibition of ROS, eventually serve as a secondary messenger for the induction of defense responses [39].

A polymer of the phenylpropanoid compound, lignin, is present constitutively in plants. However, its composition and content can vary at alternate levels on the basis of the degree of stress to which the plants are exposed. Alleviation in lignification is often observed in plants as a response to the biotic stresses experienced by them and is considered as one of the mechanisms adopted by the plant in its defense [55]. The resistance of plants to the cell-wall-degrading enzymes produced by the pathogen can be enhanced by strengthening the plant cell wall by phenolics and lignin, thereby acting as a perfunctory blockade to toxin invasion and to physical penetration toward the protoplast [56]. The transverse section of stems, exhibiting the lignin deposition in stem cells, may block the pathogen penetration, as was also observed by some researchers [57,58]. A higher lignin deposition was also observed in the present study in the defense-inducer-treated pathogen-inoculated plant, thereby indicating that the priming of plants by defense inducers before pathogen inoculation activates a faster and more pronounced defense response in them [59,60].

## 5. Conclusions

The present study very clearly demonstrates an augmented defense response in tomato plants against *Cmm* by defense inducers. The defense inducers not only elicited the antioxidant activity that caused an increased H_2_O_2_ generation but also the phenylpropanoid activity that caused an increased PAL activity followed by alleviated phenolic accumulation and lignin deposition. Moreover, there is also evidence of ROS generation as indicated by higher POX and β-1,3-glucanases activities. The increased response of all these activities in the defense-inducer treatment compared to the negative and positive controls is positively correlated with reduced disease incidence. The defense inducers in tomato crops may thus be used to enhance the defense responses toward *Cmm*.

## Data Availability

The data has been presented in the research article itself and any further data can be presented by the corresponding author on demand.
